# MALDI Spectra Database for Rapid Discrimination and Subtyping of *Mycobacterium kansasii*

**DOI:** 10.3389/fmicb.2018.00587

**Published:** 2018-04-03

**Authors:** Jayaseelan Murugaiyan, Astrid Lewin, Elisabeth Kamal, Zofia Bakuła, Jakko van Ingen, Vit Ulmann, Miren J. Unzaga Barañano, Joanna Humięcka, Aleksandra Safianowska, Uwe H. Roesler, Tomasz Jagielski

**Affiliations:** ^1^Centre for Infectious Medicine, Institute of Animal Hygiene and Environmental Health, Freie Universität Berlin, Berlin, Germany; ^2^Division 16, Mycotic and Parasitic Agents and Mycobacteria, Robert Koch Institute, Berlin, Germany; ^3^Department of Applied Microbiology, Faculty of Biology, Institute of Microbiology, University of Warsaw, Warsaw, Poland; ^4^Department of Medical Microbiology, Radboud University Medical Center, Nijmegen, Netherlands; ^5^Institute of Public Health, Ostrava, Czechia; ^6^Clinical Microbiology and Infection Control, Basurto Hospital, Bilbao, Spain; ^7^Hospital for Infectious Diseases in Warsaw, Medical University of Warsaw, Warsaw, Poland; ^8^Department of Internal Medicine, Pulmonology, and Allergology, Medical University of Warsaw, Warsaw, Poland

**Keywords:** Biotyper, matrix-assisted laser desorption ionization—time of flight mass spectrometry, microflex, *Mycobacterium kansasii*, genotypes

## Abstract

*Mycobacterium kansasii* is an emerging non-tuberculous mycobacterial (NTM) pathogen capable of causing severe lung disease. Of the seven currently recognized *M. kansasii* genotypes (I-VII), genotypes I and II are most prevalent and have been associated with human disease, whereas the other five (III-VII) genotypes are predominantly of environmental origin and are believed to be non-pathogenic. Subtyping of *M. kansasii* serves as a valuable tool to guide clinicians in pursuing diagnosis and to initiate the proper timely treatment. Most of the previous rapid diagnostic tests for mycobacteria employing the matrix-assisted laser desorption/ionization time-of-flight mass spectrometry (MALDI-TOF MS) technology focused on species-level identification. The purpose of this study was to establish MALDI-TOF MS reference spectra database for discrimination of *M. kansasii* at the genotype level. A panel of 32 strains, representatives of *M. kansasii* genotypes I-VI were selected, whole cell proteins extracted and measured with MALDI-TOF MS. A unique main spectra (MSP) library was created using MALDI Biotyper Compass Explorer software. The spectra reproducibility was assessed by computing composite correlation index and MSPs cross-matching. One hundred clinical *M. kansasii* isolates used for testing of the database resulted in 90% identification at genus-level, 7% identification at species-level and 2% identification was below the threshold of log score value 1.7, of which all were correct at genotype level. One strain could not be identified. On the other hand, 37% of strains were identified at species level, 40% at genus level and 23% was not identified with the manufacturer's database. The MALDI-TOF MS was proven a rapid and robust tool to detect and differentiate between *M. kansasii* genotypes. It is concluded that MALDI-TOF MS has a potential to be incorporated into the routine diagnostic workflow of *M. kansasii* and possibly other NTM species.

## Introduction

*Mycobacterium kansasii* is one of the leading causative agents of pulmonary and extrapulmonary infections due to non-tuberculous mycobacteria (NTM). It is also among the top six most frequently isolated NTM species across the world. The isolation rate of this pathogen is exceptionally high in Slovakia, Poland, and the UK, that is 36, 35, and 11% respectively, compared to a mean isolation rate of 5% in Europe and 4% globally (Hoefsloot et al., 2013).

The major clinical manifestations attributable to *M. kansasii* include fibro-cavitary or nodular-bronchiectatic lung disease, lymphadenitis, skin, and soft-tissue infections, and disseminated disease (van Ingen and Kuijper, 2014).

Pulmonary diseases caused by *M. kansasii* tend to cluster in specific geographical areas, such as central Europe or metropolitan centers of London, Brasilia, and Johannesburg (Hoefsloot et al., 2013). In most of the countries there is no obligation of registration of such cases, thus the true incidence of *M. kansasii* disease is largely unknown. The estimated incidence of infections due to this pathogen, reported in the general population, fall within the range of 0.3–1.5 cases per 100,000 (Santin et al., 2004; Moore et al., 2010; Namkoong et al., 2016).

As with other NTM, *M. kansasii* infections are believed to be acquired from environmental exposures rather than by person-to-person transmission. Rarely has *M. kansasii* been isolated from soil, natural water systems or animals. Instead, the pathogen has commonly been recovered from municipal tap water, which is considered to be its major environmental reservoir (Thomson et al., 2014).

The existence of five (I–V) distinct *M. kansasii* types was initially evidenced by unique DNA profiles they produced upon hybridization with the major polymorphic tandem repeat (MPTR) probe, pulsed-field gel electrophoresis (PFGE), amplified fragment length polymorphism (AFLP) analysis, and PCR restriction enzyme analysis (PCR-REA) of the *hsp65* gene (Alcaide et al., 1997; Picardeau et al., 1997). In somewhat later studies two novel types (VI and VII) have been described, with similar methods (Taillard et al., 2003). This intra-species polymorphism has important clinical and epidemiological implications. Genotypes I and II have been involved in human disease, with the former being the most prevalent, whereas the remaining genotypes have usually been non-pathogenic. Therefore, recovery of *M. kansasii* type I and II isolates from clinical specimens should raise a clinical suspicion of *M. kansasii* disease. Furthermore, *M. kansasii* type I has been found more likely to be recovered from immunocompetent individuals, whereas type II has been linked to patients with some form of immunosuppression (Taillard et al., 2003). Furthermore, despite a paucity of studies, there seems to be some genotype-specific differences in drug susceptibility profiles. For instance, clarithromycin resistance appears to be associated with *M. kansasii* type I (Li et al., 2016), while *M. kansasii* type V, contrary to all other types, shows susceptibility to ethambutol (Bakuła et al., 2018). Currently, identification of *M. kansasii* genotypes is performed by PCR-sequencing or PCR-REA of the *hsp65, rpoB*, and *tuf* genes (Telenti et al., 1993; Kim et al., 2001; Bakuła et al., 2016). These methods, though accurate and reproducible, require post-PCR manipulations, which are time-consuming and laborious. A fast, reliable and automated screening method for *M. kansasii* genotype determination is needed.

In the past two decades, MALDI-TOF MS-based species identification has been integrated into the routine diagnostic workflow in microbiology laboratories due to its rapidness, reliability, cost-effectiveness, and high throughput. A prerequisite for MALDI-TOF MS profiling is a robustly established reference spectra database. The growing accessibility of MALDI-TOF MS instruments along with spectra pattern matching software databases made that the interest in this technology and its applications across research and diagnosis has exponentially increased (Wattal et al., 2017).

In this study, a commercially available software package, MALDI Biotyper (Bruker Daltonics) was used for creation of genotype-specific reference database of *M. kansasii*.

## Materials and methods

### Mycobacteria strains and isolates

A total of 32 strains, representing I-VI genotypes of *Mycobacterium kansasii* were used to establish a MALDI-TOF MS spectra reference database (Table 1). In addition, 100 clinical *M. kansasii* isolates were used for evaluation of the database. The species identity of these isolates was confirmed with the high-pressure liquid chromatography (HPLC). Genomic DNA was extracted with the AMPLICOR Respiratory Specimen Preparation Kit (Roche, Basel, Switzerland). Genotyping was performed upon PCR-REA of *hsp65* and *tuf* genes, as previously described (Telenti et al., 1993; Bakuła et al., 2016).

**Table 1:** *M. kansasii* strains and their genotypes utilized for generation of reference spectra.

**S. No**	**Strain designation**	**Genotype**	**Source**	**NTM disease**	**Country**
1	*M. kansasii* ATCC12478^T^	I	No data	Yes	USA
2	*M. kansasii* ATCC25221^T^		Sputum	Yes	Germany
3	*M. kansasii* NLA001000449		Sputum	Yes	Netherlands
4	*M. kansasii* NLA001000521		Bronchoalveolar lavage fluid	Yes	Netherlands
5	*M. kansasii* 1502/11		Bronchoalveolar lavage fluid	Yes	Poland
6	*M. kansasii* 803/13		Bronchial washing	Yes	Poland
7	*M. kansasii* 5482/08		Bronchial washing	Yes	Poland
8	*M. kansasii* 305/01		Sputum	No	Poland
9	*M. kansasii* 1010001495^E^		Water	–	Czech Republic
10	*M. kansasii* 10130/12		No data	Yes	Germany
11	*M. kansasii* 4911/16		Sputum	Yes	Germany
12	*M. kansasii* 8034/16		Bronchoalveolar lavage fluid	Yes	Germany
13	*M. kansasii* 8050/16		No data	Yes	Germany
14	*M. kansasii* 8091/16		No data	Yes	Germany
15	*M. kansasii* 8112/16		Bronchoalveolar lavage fluid	Yes	Germany
16	*M. kansasii* 8617/16		Sputum	Yes	Germany
17	*M. kansasii* B11063838	II	Bronchoalveolar lavage fluid	No	Netherlands
18	*M. kansasii* B11073207		Sputum	No	Netherlands
19	*M. kansasii* NLA001001128		Bronchoalveolar lavage fluid	No	Netherlands
20	*M. kansasii* 2193/11		Bronchial washing	No	Poland
21	*M. kansasii* 1010001469^E^		Water	–	Italy
22	*M. kansasii* 8190/16		Tracheal secretion	Yes	Germany
23	*M. kansasii* 8709/16		Bronchial secretion	Yes	Germany
24	*M. kansasii* 1010001468^E^	III	Soil	–	Belgium
25	*M. kansasii* 8197/16		No data	Yes	Germany
26	*M. kansasii* 101		No data	Yes	Germany
27	*M. kansasii* 1010001458^E^	IV	Tap water	–	Germany
28	*M. kansasii* 1010001454^E^	V	Tap water	–	Germany
29	*M. kansasii* 1010001493^E^		Water	–	Netherlands
30	*M. kansasii* 6097/16		Bronchial secretion	Yes	Germany
31	*M. kansasii* NLA001001166	VI	Sputum	No	Netherlands
32	*M. kansasii* NTM G7 Lu1		Gundi, lung tissue	Yes	Germany

T *Type strain*,

E *Environmental isolate*.

### Culture and protein extraction

A single bacterial colony was picked from Löwenstein-Jensen (L-J) slant and used for inoculation of 10 mL of Middlebrook 7H9 medium supplemented with glycerol and OADC (10%) (Becton-Dickinson, Franklin Lakes, USA). The bacteria were cultured at 37°C with shaking for 7 days. Each strain/isolate was cultured independently for three independent replicates. The cells were harvested by brief centrifugation (4,500 rpm, 2 min) and killed by suspending in 300 μL of deionized water and 900 μL of absolute ethanol. After centrifugation (12,000 rpm, 2 min) at room temperature (RT), excessive ethanol was discarded and the samples were air-dried completely. The cell pellet was dissolved in 50 μL of 70% formic acid and 50 μL acetonitril. The samples were then subjected to sonication on ice for 1 min (cycle, 1.0; amplitude, 100%) using a sonicator (UP100H, Hielscher Ultrasound Technology, Teltow, Germany). The suspension was centrifuged at 12,000 rpm for 2 min at RT, and 1.0 μL of the clear supernatant was spotted in triplicate onto the MALDI target (MSP 96 target polished steel (MicroScout Target) plate, Bruker Daltonik, Bremen, Germany). Following air drying each sample was overlaid with 1.0 μL of saturated α-cyano-4-hydroxycinnamic acid matrix solution and allowed to dry completely prior to MALDI-TOF measurement.

### MALDI measurements

Bruker MALDI Microflex LT (Bruker Daltonics, Bremen, Germany) with a linear positive mode of spectra acquisition, at a laser frequency of 20 Hz was used for the measurements. A broad m/z range (2,000–20,000 kDa) of spectra was automatically acquired using the AutoXecute acquisition control software (Flex control 3.0, Bruker Daltonics, Leipzig, Germany) with the following instrument settings: ion source 1 at 20 kV, ion source 2 at 16.7 kV, lens at 6 kV, extraction delay time of 150 ns, 240 laser shots/spot in 40 shot steps (random walk movement), initial laser power of 45%, maximal laser power of 55%, laser attenuation Offset of 46% and at the range 30%). The bacterial test standard (BTS) mixture covering the mass range between 2,000 and 20,000 Da (BTS, Bruker Daltonics, Bremen, Germany), was used for external calibration.

### MSP library construction

The spectra quality and reproducibility were assessed by using Flex analysis 3.0 software (Bruker Daltonics, Bremen, Germany). For every strain/isolate, 27 spectra representing three independent culturing and three technical replicates and 3 measurements per replicate were collected. The raw spectra were loaded in the MALDI Biotyper Compass Explorer 4.1 and matched against the commercial BDAL database—updated version 6.0.0.0 with 6,903 Main Spectra (MSP) entries (including 75 for mycobacteria with seven genotype undefined *M. kansasii* entries)—plus the in-house entries of *Staphylococcus intermedius* group of microorganisms (60 entries) (Murugaiyan et al., 2014) and microalgae *Prototheca* species (23 entries) (Murugaiyan et al., 2012). The spectra preprocessing parameters included mass adjustment (lower bound- 3,000, upper bound- 15,000; resolution-1 and spectra compressing factor- 10), smoothing (Savitzky-Golay algorithm with a frame size of 25 Da), baseline correction (multipolygon with search window 5 Da and number of runs 2), normalization (maximum norm), and peak detection (spectra differentiation with maximum peaks of 100, signal-to-noise ratio 3 and threshold of 0.001). Main spectrum profiles (MSPs) were created for each strain using the following parameters: maximum mass error for each single spectrum 2,000, mass error for the MSP 100, peak frequency minimum 70%, and maximum peak number 70. The details of the newly created reference spectra will be listed at the MALDI-UP, an online platform for the MALDI-TOF mass spectra (Rau et al., 2016) to facilitate exchange of the reference spectra as btmsp files with users from other laboratories.

### Statistical evaluations

To evaluate the spectral variation (similarity) between the spectra sets acquired from strains of all genotypes, the Composite Correlation Index (CCI) was calculated with MALDI Biotyper Compass Explorer 4.1. All measured spectra were loaded and CCI was computed using the following settings, mass lower bound 3,000, mass upper bound 12,000, resolution 4, and CCI parameter interval of 8. CCI value nearing 1.0 indicates the complete congruence and 0.0 – complete deviation within the spectra sets.

Following the creation of spectral reference database, the genotype discrimination limits were assessed by crosswise comparison by matching the newly created *M. kansasii* genotype-specific MSPs with an augmented BDAL commercial database described earlier. The resulted log score values of the first hits were imported into MS excel and conditional formatting, in accordance to the manufacturer's recommended cutoff log score values, was applied to generate a heat-map. The score-oriented MSP dendrogram (distance level: correlation and linkage: average) was also calculated using the MALDI Biotyper Compass Explorer 4.1 software to explore the closeness of the genotypes in terms of arbitrary distance level.

### Blind coded sample testing

For validation purposes, 100 clinical *M. kansasii* isolates and single reference strains of *M. conspicuum, M. marinum, M. szulgai*, and *M. gastri* were subjected to MALDI-TOF MS identification, by comparing with the database before and after addition of genotype-specific MSPs (Supplementary Table 1). The isolates used for validation, had previously been identified, as specified above and blind coded. The log score values for species discrimination, recommended by the manufacturer, were applied i.e., 0–1.699 indicating “no reliable identification”, 1.7–1.999 indicating a “probable genus identification”, 2.0–2.299 indicating a “secure genus identification and probable species identification” (PSI), and 2.300–3.000 indicating a “highly probable species identification.”

## Results

A total of 32 strains representing genotypes I-VI of *M. kansasii* were used to establish *M. kansasii* genotype-specific reference spectra library. The choice of the strains was based on the availability of strains representing a given genotype and strains of genotype VII were not available in any of the culture collections. Each strain was cultured independently three times, each culture-derived extract was spotted three times, and each spot was measured three times on MALDI-TOF MS to generate 27 spectra in total per strain. Altogether, 864 spectra within the mass range of 2,000–20,000 Da were acquired. The spectra quality in terms of peak presence and intensity were assessed using Flex analysis software. The flat line spectra and low-intensity spectra were replaced by new measurement of the same spots or fresh spotting of the same protein extract.

**Figure 1:**
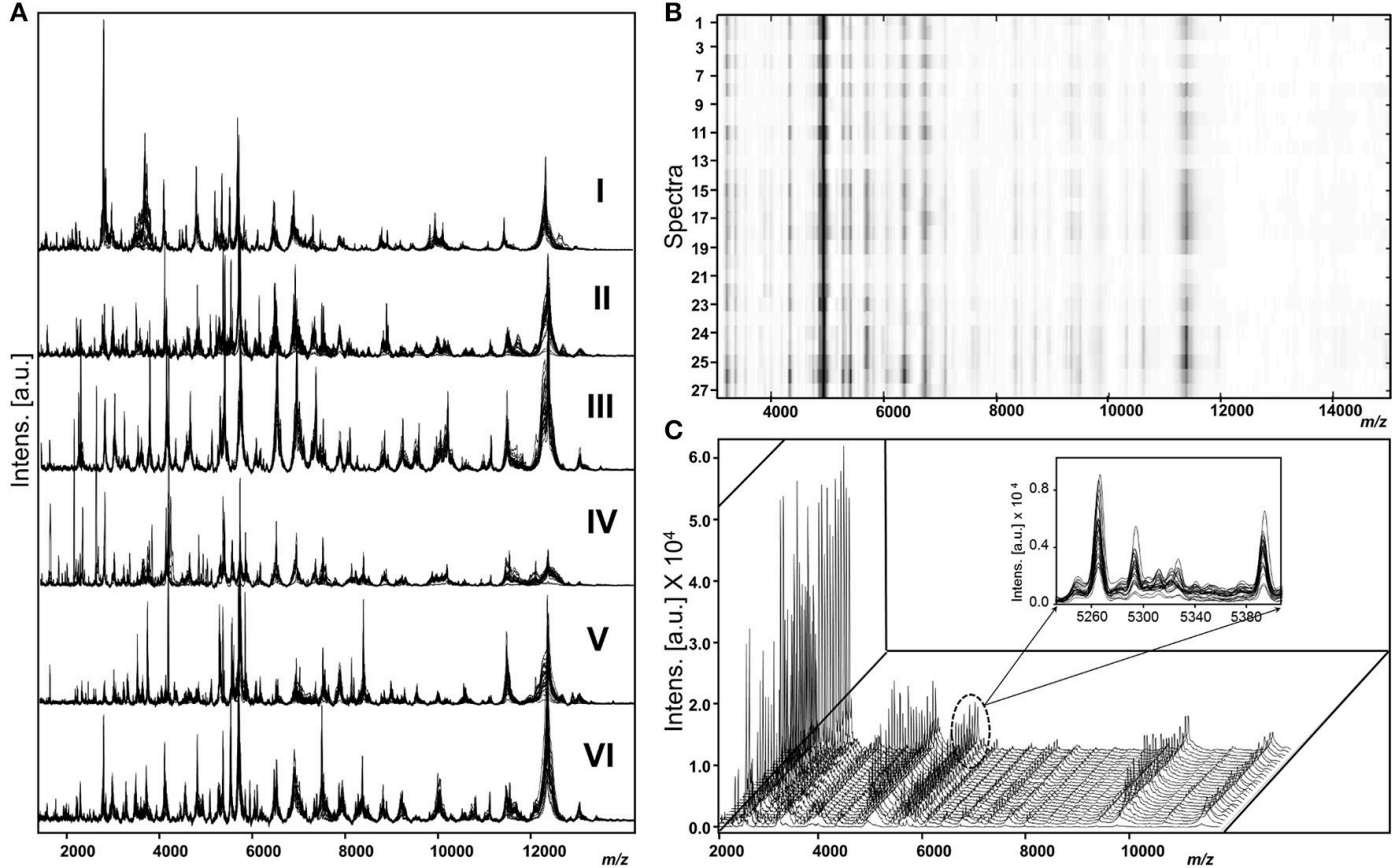
Representative raw spectra of *M. kansasii* genotypes **(A)** overlaid view of spectra sets (*n* = 27) of genotype I (strain 1010001495), genotype II (Strain 2193/11), genotype III (strain1010001468), genotype IV (strain 1010001458), genotype V (strain 6097/16), and genotype VI (strain NLA 001001166). **(B)** Heat map view of spectra acquired from a single strain (genotype I strain 1010001495). **(C)** Stacked view indicating the uniformity among spectra acquired from a genotype I strain 1010001495 and highlighted overlaid view as an inserted figure.

As shown in Figure 1, different genotypes displayed distinct peak patterns and the spectral sets of each strain were fully reproducible and comparable.

**Figure 2:**
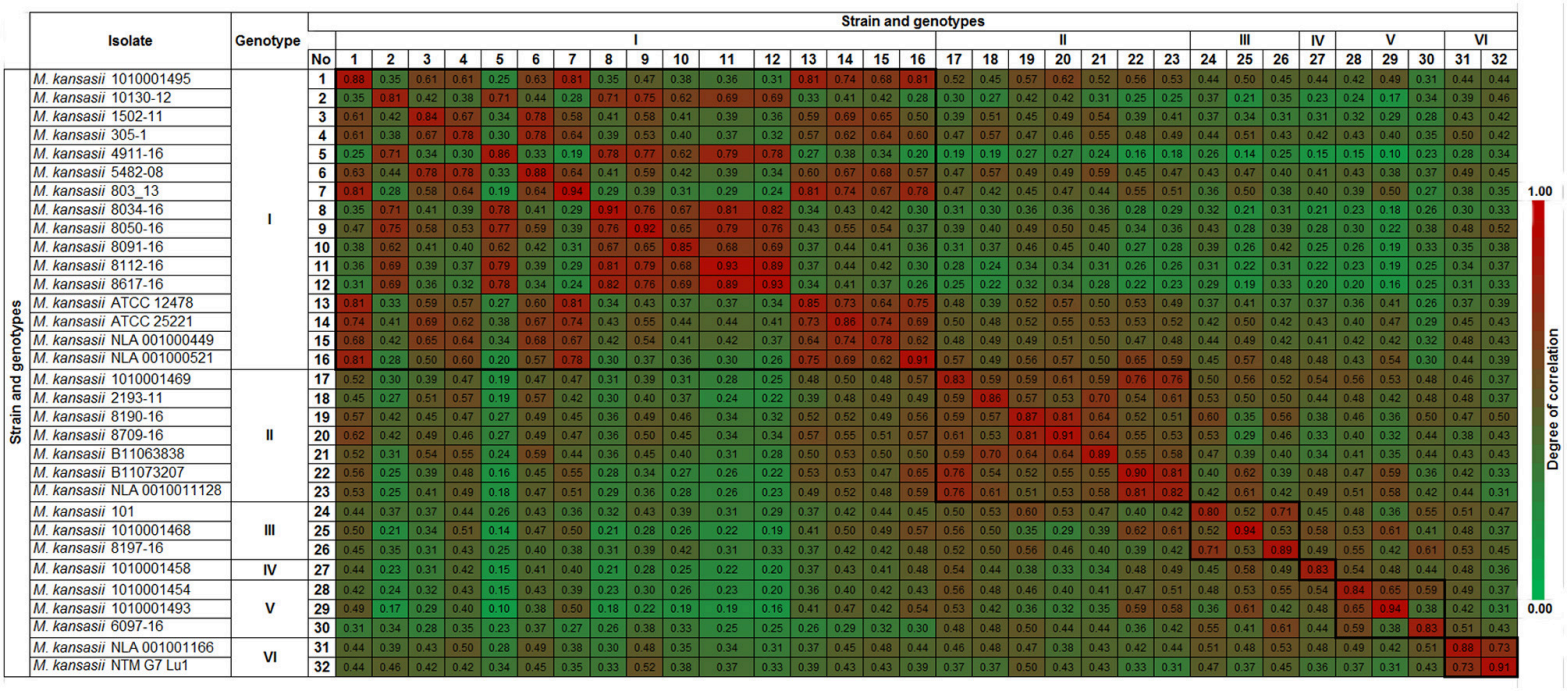
Composite Correlation Index (CCI) matrix as calculated using Biotyper RTC software with the following parameter settings: 3,000 lower bound, 15,000 upper bound, mass range resolution of 4 and number of interval set at 4. The CCI values were extracted and the displayed image was redrawn using conditional formatting option in MS Excel. The CC value nearing 1.0 indicates the congruence among the measured spectra sets and 0 represents completely different spectra.

The spectra of the same strains showed a high level of similarity, with the CCI value ranging from 0.78 to 0.94 (Figure 2). Genotype I displayed CCI within the range of 0.78 to 0.99 while it was between 0.82 and 0.91 for genotype II, 0.80 to 0.94 for genotype III, 0.83 for a single genotype IV strain, 0.83 to 0.94 for genotype V, and 0.88 to 0.91 for genotype VI. The resulted CCI values reflected the similarities of the measured spectra sets. The CCIs between different genotypes displayed lower values indicating the possibility for spectra fingerprinting of genotype level discrimination.

The created *M. kansasii* genotype MSPs were then matched to the augmented BDAL database described earlier to generate the cross-matching result. Then, the first hits log score values were imported in MS excel and a heat map was generated using MS excel conditional color formatting option following the manufacturer's cut-off values (Supplementary Figure 1). The MSPs of any given strain matched 100% with themselves, as indicated by the log score of 3.0, and had high matching scores with the other strains within a genotype. The seven strains without any genotype information included in the original version of the manufacturer's database, displayed higher score values among each other, although the MSPs had been configured upon a much lower number of spectra. The manufacturer's MSPs displayed log score values of >2.0 with several strains of newly added genotype I and borderline or genus-level matching was observed with genotype III, IV, and V strains. One of the manufacturer's MSP (S.No. 37) did not match any of the newly introduced genotype-specific MSPs.

**Figure 3:**
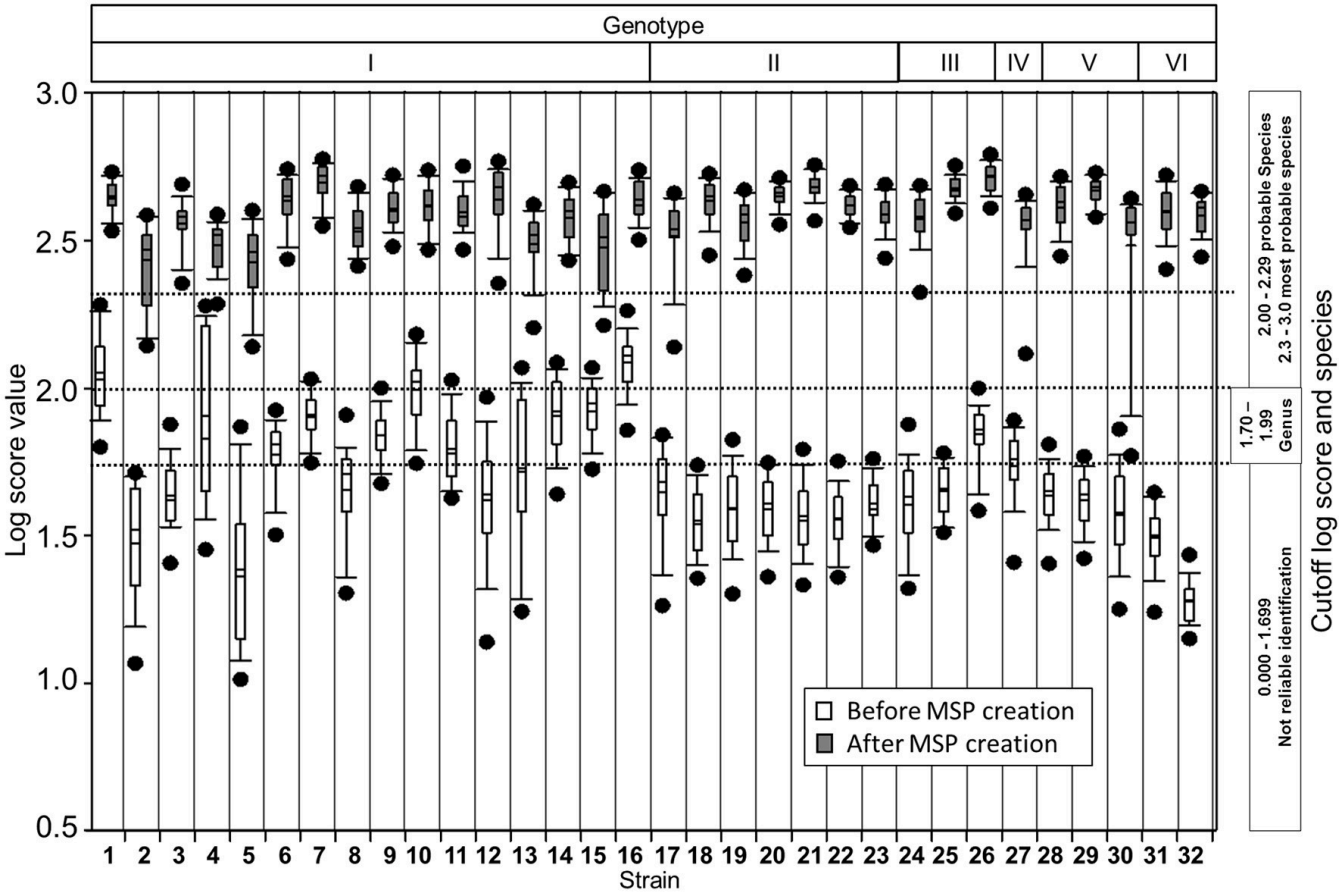
Box plot of log score values before and after creation of in-house database for *M. kansasii* genotypes. Plots show mean and median (horizontal lines within each boxes), 5th and 95th percentiles (whiskers) and outliers (black dots) for each set of the measured spectra. Log score values compared to the database before (white boxes) and after (gray) creation of genotype-specific reference spectra indicate improvement in the identification confidence.

In addition, the individual spectra used for MSP creation were matched first with the manufacturer's database (BDAL database-plus in-house entries of *Staphylococcus intermedius* group and microalgae *Prototheca* species) and then after introduction of our genotype-specific MSPs. This enhanced considerably the overall identification confidence and identification at the genotype level with first hits always being the same strain (Figure 3).

**Figure 4:**
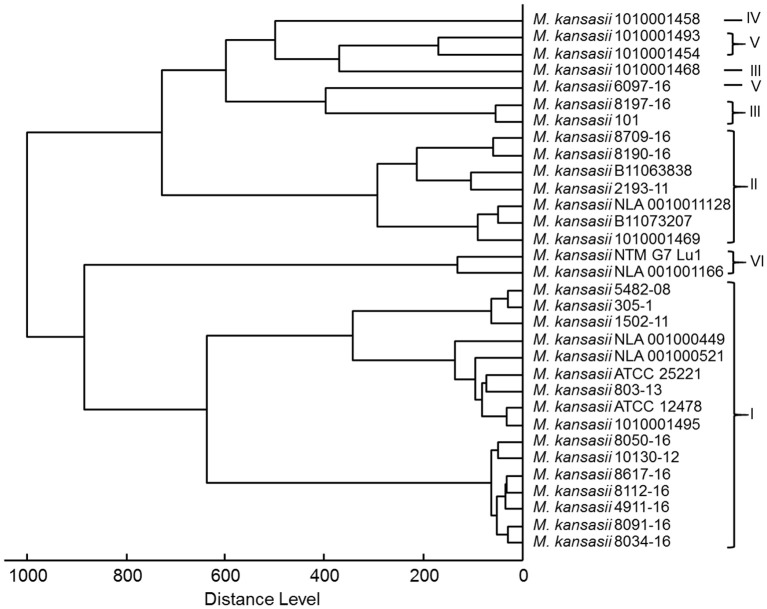
Score-oriented dendrogram of genotype-specific MSPs (distance measure was set at correlation and linkage was set at complete).

As shown in Figure 4, a score-oriented dendrogram demonstrated two major clusters. The first cluster contained all genotype I strains and two genotype VI strains. The second cluster accommodated all type II, III, IV, and V strains.

**Figure 5:**
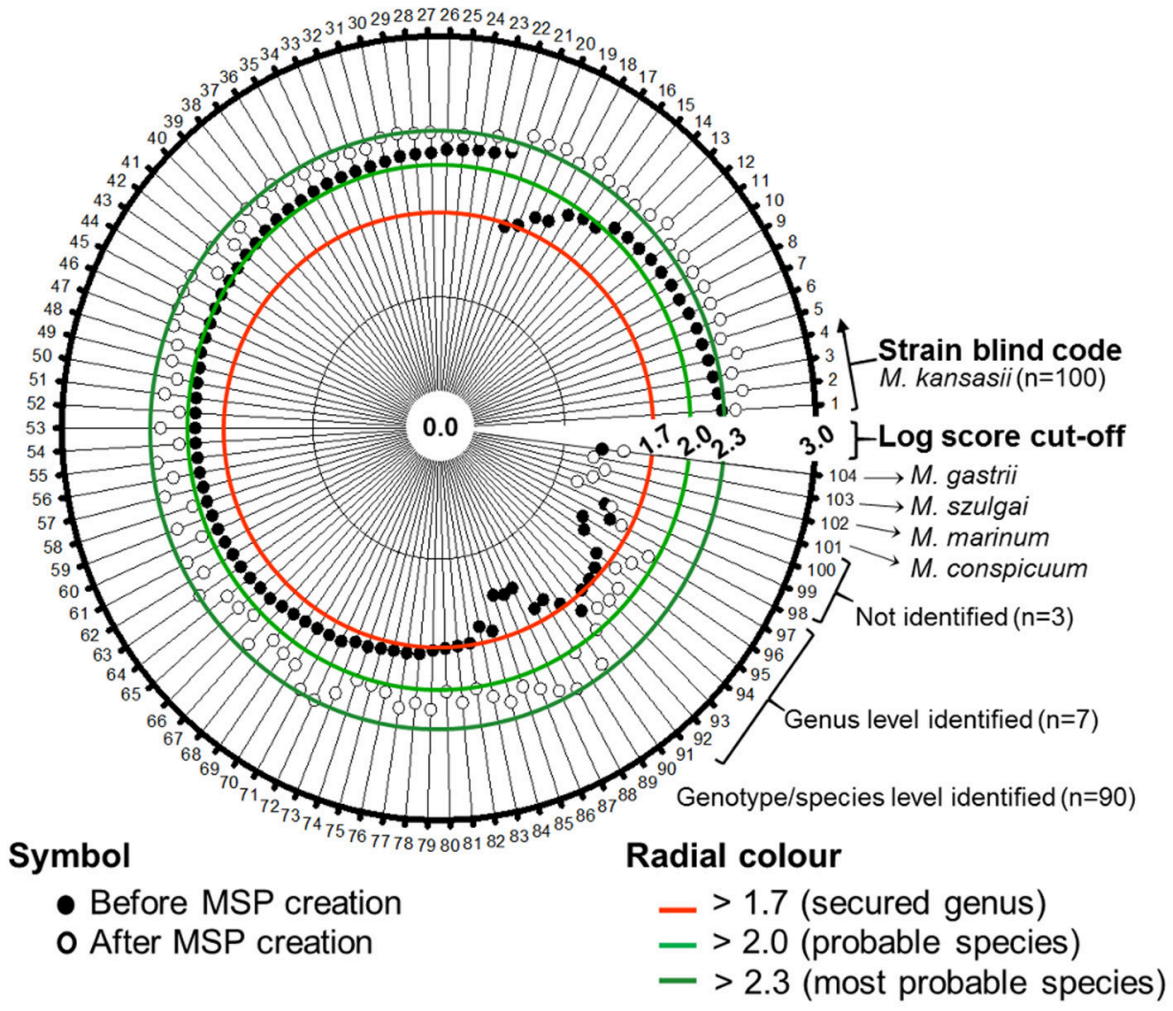
Polar plot was constructed to show the enhancement of log score values of blind-coded *M. kansasii* isolates (*n* = 100). No difference in the log score values were observed for the four non-*M. kansasii* species included in the database evaluation.

The augmented genotype-specific reference database was tested with 104 blind-coded samples, representing clinical isolates of *M. kansasii* (*n* = 100) and single strains of *M. conspicuum, M. gastri, M. marimum*, and *M. szulgai*, to find any cross-matching with *M. kansasii* database. The identification rates were significantly higher for the expanded MALDI Biotyper database when compared with the original library (Table 2). In Figure 5, a shift of identification confidence is shown for all 100 blind-coded *M. kansasii* samples (Supplementary Table 1). The augmented database resulted in an overall identification success rate of 97% isolates identified at the species/genotype level, among which 7 isolates, representatives of genotype I, were identified at the genus level. Two genotype I isolates could not be identified despite the first hit as the correct genotype but with log score value < 1.7. One isolate (*M. kansasii* 17.14) did not display any improvement in the log score value despite the database augmentation. The results also indicated that, 89 strains belonged to genotype I (19-SI, 63-PSI, and 7-PGI), 4 – to genotype II, and 2 – to genotype III. Single isolates were recognized as representing genotypes IV and V. Whereas the MALDI Biotyper using the manufacture's BDAL database displayed “Not reliable identification” for 15% isolates of genotype I and six other genotype isolates. Two genotype II isolates were identified only as *Mycobacterium* sp.

**Table 2:** Identification results of 100 blind-coded *M. kansasii* and one each of *M. conspicuum, M. marinum, M. szulgai*, and *M. gastri* strains using MALDI Biotyper Explorer Compass 4.1 reference database and after augmentation of genotype-specific reference database.

**Identification result**	**Score**	**MALDI Biotyper explorer compass 4.1**	**Augmented genotype-specific database**
Not reliable identification (NRI)	<1.7	23 (23%)	3 (3%)
Probable genus identification' (PGI)	1.7–2.0	40 (40%)	7 (7%)
Secure genus identification and probable species identification (PSI)	2.0–2.3	37 (37%)	69 (69%)
Highly probable species identification(SI)	>2.3	0	21 (21%)

## Discussion

MALDI-TOF MS profiling has been successfully employed in various commercial software packages allowing the identification of a wide array of *Mycobacterium* species, including *M. kansasii*. The discrimination of different *M. kansasii* genotypes is useful for the clinical categorization of *M. kansasii* infection, however, it has never been attempted by MALDI-TOF analysis (Pignone et al., 2006; El Khechine et al., 2011; Saleeb et al., 2011; Balada-Llasat et al., 2013; Chen et al., 2013; Machen et al., 2013; Mather et al., 2014; Mediavilla-Gradolph et al., 2015; Rodriguez-Sanchez et al., 2015; Wilen et al., 2015; Ceyssens et al., 2017; Leyer et al., 2017). *M. kansasii* genotype level information is still not included in the commercial reference database package, MBT Mycobacteria library. Previously, identification of *M. kansasii* with low spectral scores was linked to the intra-species heterogeneity, and supplementation of the database with additional strains of *M. kansasii* was proposed to remedy this problem (El Khechine et al., 2011; Saleeb et al., 2011). Responding to this necessity, this study established MSPs for all *M. kansasii* genotypes (I–VI), except for the genotype VII (Taillard et al., 2003), for which strains could not be retrieved in any of the culture collection, which could not be retrieved from any culture collections. It has to be noted, however, that given the paucity of genotype II-VI strains, differentiation of these types may still pose certain difficulties, and much more strains would be needed to verify the genotype-specificity of the MALDI-TOF profiles established.

Inspection with Flex analysis software, the spectral profiles of the *M. kansasii* genotypes clearly showed their disparateness. At the same time, a high intra-genotype reproducibility of the spectra was demonstrated. The spectra sets acquired from the same strains and from strains representing the same genotypes were highly consistent, as evidenced with the computed CCI values.

The cross identification of the established MSPs together with seven MSPs originally deposited in the manufacturer's database resulted in a log score value of 3.0 indicating matching of the 70 most frequently encountered peaks of the species or genotypes. When compared against the manufacturer's database, with exception of genotypes I and II, whose spectra matched those of *M. kansasii*, all other genotypes produced spectra ascribable to merely *Mycobacterium* genus level. It clearly proved that the existing database is not sufficient for genotype-level discrimination of *M. kansasii*. Special mycobacteria library with lowered threshold log score values of 1.6 and 1.8 was implemented for genus and species identification. It was later reported that the log score values for *M. kansasii* were unchanged with the improved mycobacteria library (Rodríguez-Sánchez et al., 2016). In the present study, augmentation of genotype-specific MSPs resulted in an enhanced log score value, which indicates that the identification confidence improvement can be mainly achieved through inclusion of genotypically well-defined strains.

The grouping, as shown on score-oriented dendrogram, of genotypes I, II, and VI in three distinct clusters supports the potential of MALDI-TOF to easily differentiate between these three types. Whereas identification of genotypes III, IV, and V may pose certain difficulties, since they all belonged to a single cluster. Inclusion of additional strains of these genotypes may produce better identification scores.

The blind coded *M. kansasii* samples tested with augmented genotype specific MSPs resulted in an overall identification success rate of 97%. Eighty-eight (98%) out of 91 blinded coded samples of genotype I gave a genotype match, despite the fact that log score values for seven strains were within the genus level cutoff (1.7–1.99). Four strains of genotype II, two of genotype III, and single strains of genotype IV and V were used for blind coded study, which resulted in successful identification of all strains at the species level, otherwise, except one genotype II strain, all these strains resulted in log score value < 1.7 before the MSP augmentation. Despite an exhaustive search through culture collections of *M. kansasii* strains, genotype II–IV strains were seriously underrepresented. According to previous studies, *M. kansasii* involved in human disease belong almost exclusively to genotypes I and II, with the former accounting for 42–100% of the clinical isolations (Alcaide et al., 1997; Bakuła et al., 2013). The other types (III-VII), only sporadically recovered, are predominantly of environmental origin (Alcaide et al., 1997; Santin et al., 2004). The reason why seven isolates could be identified at the genus level only, and three isolates unidentifiable at all can hardly be found. We propose that the addition of spectra from genotype II-VI from various geographical regions could enable accurate and rapid differentiation of these genotypes.

In conclusion, unique, reproducible spectra for six genotypes of *M. kansasii* were established. The expansion of the database with reference spectra resulted in successful species- and genotype-level identification despite the limited number of genotype II–VI strains. Overall, we believe that MALDI-TOF MS can easily be employed for efficient genotype-level discrimination of *M. kansasii* and that further augmentation of spectral patterns might enable fast and accurate diagnosis in future.

## Author contributions

JM, TJ conceived and designed the work, acquired the data and wrote the manuscript. AL, EK, VU, MU, JH, and AS collected the strains and performed species-level identification. ZB performed species confirmation and genotype-level identification. JI and UR critically revised the manuscript. All authors had read and approved the submission version of the manuscript.

### Conflict of interest statement

The authors declare that the research was conducted in the absence of any commercial or financial relationships that could be construed as a potential conflict of interest. The reviewer AVDB declared a past co-authorship with one of the authors JI to the handling Editor.

## Supplementary material

Supplementary Figure 1 MSP matching matrix was created by matching with *M. kansasii* genotype-specific MSPs augmented BDAL database. Color coded as per the manufacturer's recommendation on log score cutoff values.

Supplementary Table 1 Blind coded isolates testing. Manufacturers recommended interpretation of score values were followed: from 0 to 1.699 indicates “no reliable identification” (Red); 1.7 to 1.999 indicate a “probable genus identification” (yellow); 2.0 to 2.299 indicate a “secure genus identification and probable species identification” (light green), and values from 2.300 to 3.000 indicate a “highly probable species identification” (intensive green).

## Abbreviations

MALDI-TOF MS: Matrix-assisted laser desorption ionization-time-of-flight mass spectrometry
MSP: main spectra libraries.
