# Supplementary figures and images for: MALDI Spectra Database for Rapid Discrimination and Subtyping of Mycobacterium kansasii
Source: Front Microbiol. 2018 Apr 3;9:587. doi: 10.3389/fmicb.2018.00587 (PMC5893902; doi:10.3389/fmicb.2018.00587)

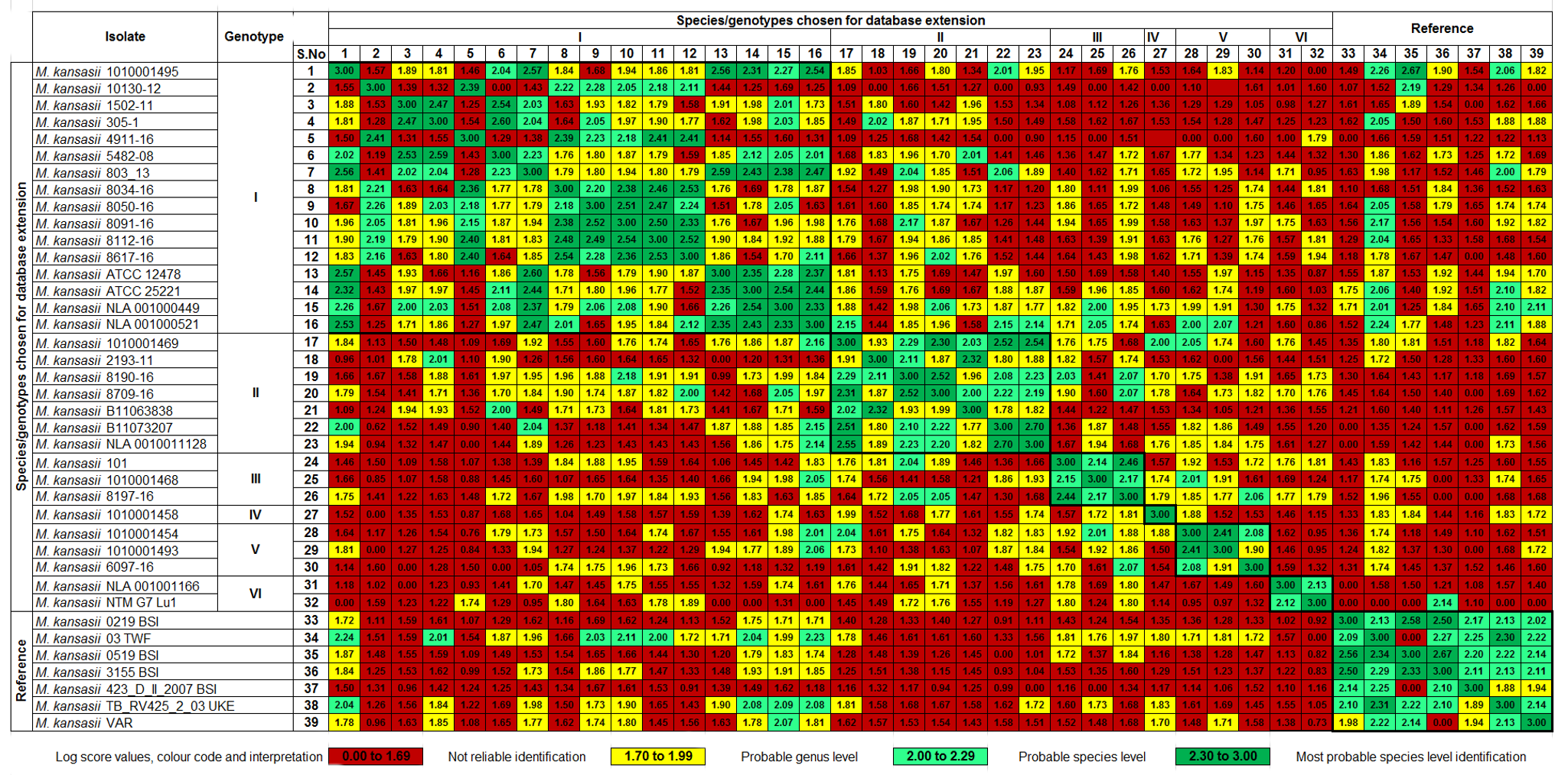

Supplement: Supplementary Figure 1 — MSP matching matrix was created by matching with M. kansasii genotype-specific MSPs augmented BDAL database. Color coded as per the manufacturer's recommendation on log score cutoff values. [file Image1.TIFF]
